# The role of Vps4 in cancer development

**DOI:** 10.3389/fonc.2023.1203359

**Published:** 2023-06-19

**Authors:** Li Juan Huang, Shi Tong Zhan, Yu Qin Pan, Wei Bao, Ye Yang

**Affiliations:** ^1^Obstetrics and Gynecology Department, Shanghai General Hospital, Shanghai Jiao Tong University School of Medicine, Hongkou, Shanghai, China; ^2^Surgical Department, Shanghai General Hospital, Shanghai Jiao Tong University School of Medicine, Hongkou, Shanghai, China

**Keywords:** Vps4, cancer, mechanisms, exosome, cell death

## Abstract

VPS4 series proteins play a crucial role in the endosomal sorting complexes required for the transport (ESCRT) pathway, which is responsible for sorting and trafficking cellular proteins and is involved in various cellular processes, including cytokinesis, membrane repair, and viral budding. VPS4 proteins are ATPases that mediate the final steps of membrane fission and protein sorting as part of the ESCRT machinery. They disassemble ESCRT-III filaments, which are vital for forming multivesicular bodies (MVBs) and the release of intraluminal vesicles (ILVs), ultimately leading to the sorting and degradation of various cellular proteins, including those involved in cancer development and progression. Recent studies have shown a potential relationship between VPS4 series proteins and cancer. Evidence suggests that these proteins may have crucial roles in cancer development and progression. Several experiments have explored the association between VPS4 and different types of cancer, including gastrointestinal and reproductive system tumors, providing insight into the underlying mechanisms. Understanding the structure and function of VPS4 series proteins is critical in assessing their potential role in cancer. The evidence supporting the involvement of VPS4 series proteins in cancer provides a promising avenue for future research and therapeutic development. However, further researches are necessary to fully understand the mechanisms underlying the relationship between VPS4 series proteins and cancer and to develop effective strategies for targeting these proteins in cancer therapy. This article aims to review the structures and functions of VPS4 series proteins and the previous experiments to analyze the relationship between VPS4 series proteins and cancer.

## 1 Introduction

Aberrant expression or sporadic mutations in the endosomal sorting complex required for transport (ESCRT) have been observed in an increasing number of cancers (1), suggesting a potential link to the ESCRT pathway (2). Vacuolar protein sorting 4 (VPS4) is an ATPase that plays a crucial role in driving membrane constriction (3), making it a key functional component in the ESCRT pathway (4). In this article, we review the structures and functions of VPS4 proteins and analyze previous experiments to provide a comprehensive understanding of their role in cancer.

## 2 The structures and functions of VPS4

### 2.1 Overview of ESCRT

ESCRT is a hetero-multimeric protein machinery mediating inverse membrane remodeling (5). ESCRT proteins assemble on the cytosolic or nucleoplasmic side of the neck of the forming involution and work together with the ATPase VPS4 to facilitate membrane scission or sealing (6). The functions of ESCRTs can be generalized as follows: cytokinetic abscission, plasma membrane repair, vesicle budding from plasma membrane, endosomal sorting and ILV biogenesis (7), autophagy, repair of endo-lysosomal membranes (3, 8).

**Figure 1:**
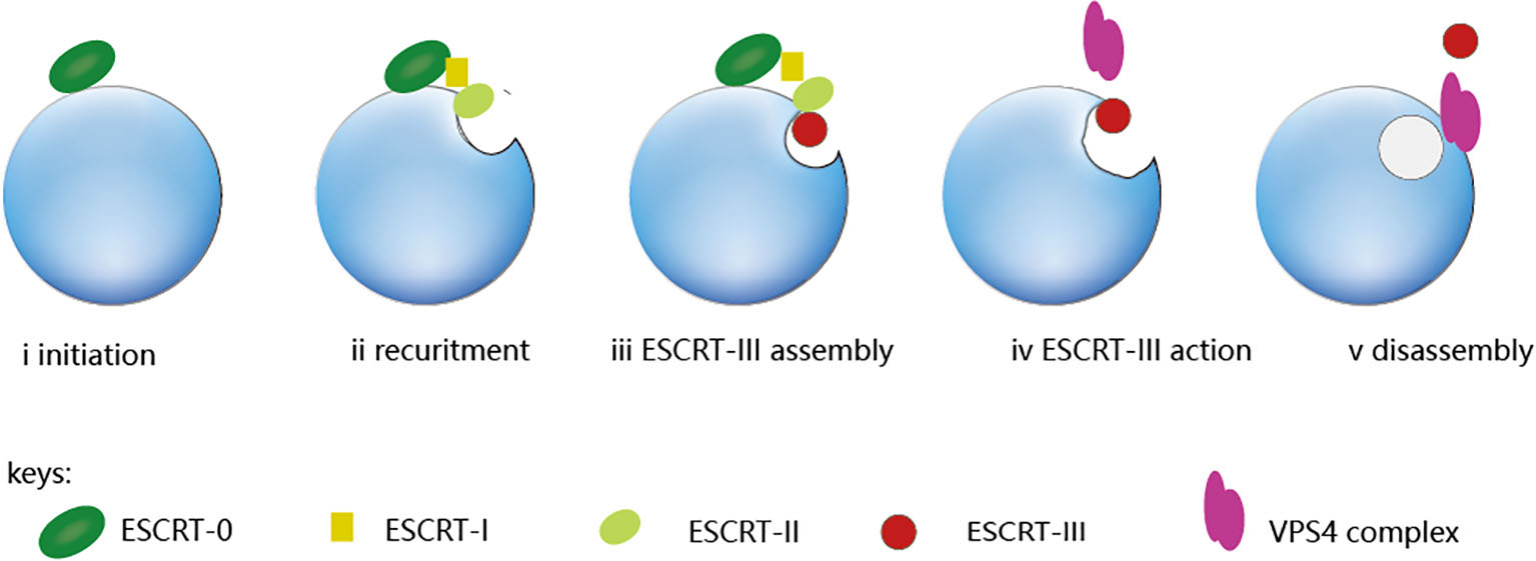
The ESCRT pathway. From the ESCRT protein standpoint, the process is initiated by ESCRT-0, which engages with ubiquitinated cargo (i). The ESCRT-I and ESCRT-II complexes bind with cargo and each other, which creates an ESCRT-cargo-enriched zone (ii) and is subsequently sequestered and sorted by ESCRT-III (iv and v). The nucleation site for ESCRT-III assembly is provided by the ESCRT-II complex. (iii), which drives vesicle budding (iv) and is subsequently disassembled by the Vps4 complex (v).

ESCRT is a complex of four subunits including ESCRT-0, ESCRT-I, ESCRT-II, and ESCRT-III. VPS4, as an ATPase, is required for the disassembly of ESCRT-III polymer (9). ESCRT-0 is responsible for identifying and clustering substrates on membranes, making it the driving force for cargo clustering in the ESCRT pathway (3); ESCRT-I and II induce membrane bud formation and cargo positioning and then localize to the necks of membrane buds to recruit ESCRT-III subunits and activate scission; ESCRT-III shears the top of the budding body and releases the vesicles. This polymerization sequence drives membrane deformation and fission (10), which requires energy from AAA+ ATPaseVps4. The ESCRT-III subunits are disassembled through conformational changes induced by the VPS4 ATPases, which harness the energy derived from ATP hydrolysis (11). **Figure 1** illustrates the complete ESCRT pathway.

**Figure 2:**
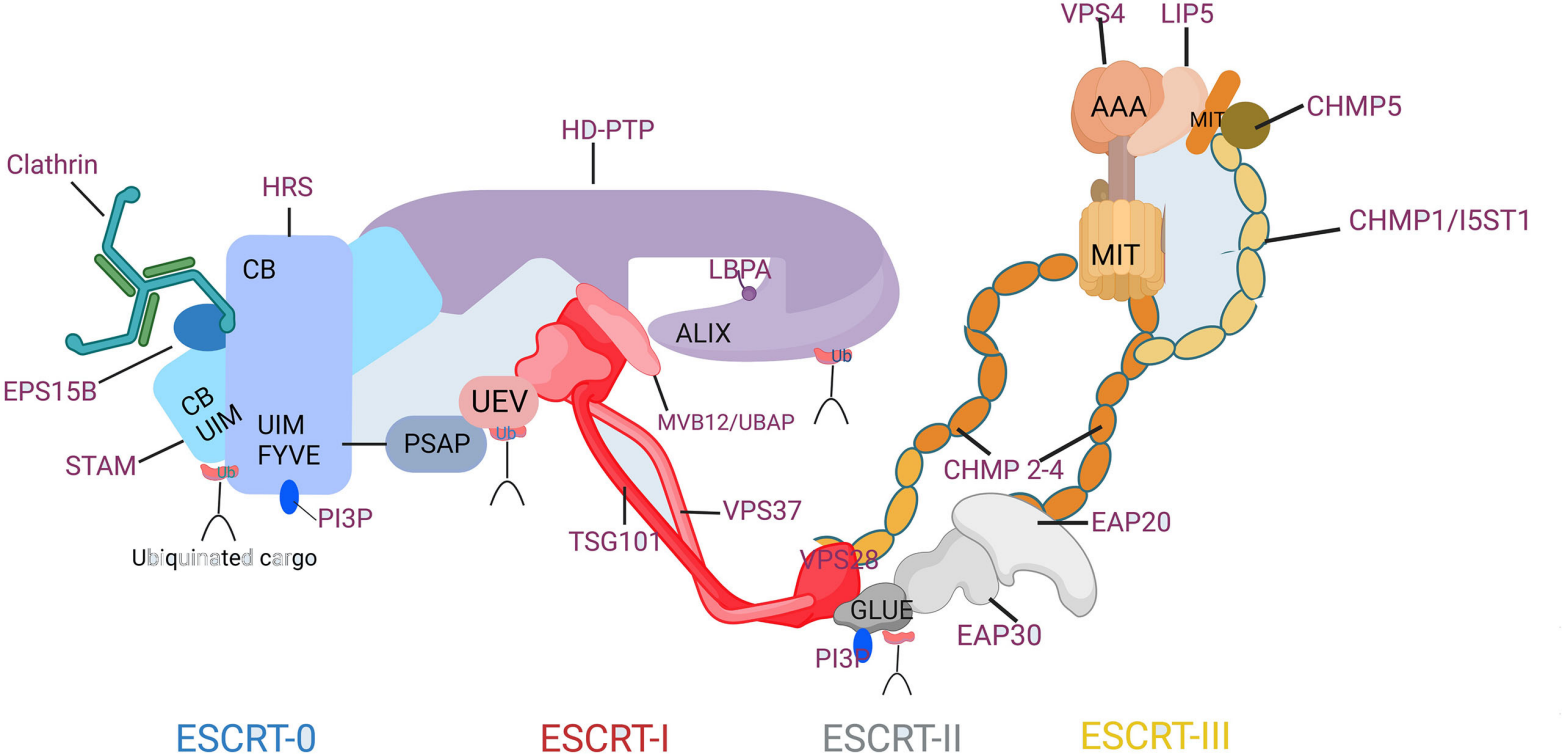
Composition and Molecular Interactions of the ESCRT Machinery. CB, clathrin-box motif; FYVE, Fab1p/YOTB/Vac1p/EEA1 domain; GLUE, GRAM-like ubiquitin in EAP45 domain; MIT, microtubule interacting and transport domain; Ub, ubiquitin; UEV, ubiquitin conjugated enzyme E2 variant; UIM, ubiquitin-interacting motif.

The initiation of endosomal ESCRT activity is facilitated by the binding of the ESCRT-0 protein HRS(Hepatocyte growth factor-regulated tyrosine kinase substrate) to the endosomal lipid, phosphatidylinositol 3-phosphate (PI3P), as illustrated in **Figure 2**(Created with BioRender.com). HRS is a protein that plays a crucial role in endosomal sorting and trafficking of ubiquitinated cargo. Together with the ESCRT-0 subunit STAM (Signal Transducing Adaptor Molecule), HRS binds to ubiquitinated cargo and to the coat protein clathrin, which helps to concentrate ESCRT-0 in endosomal microdomains. Other accessory proteins such as Eps15B, an endocytic adaptor protein that interacts with both HRS and clathrin and plays a role in the formation of clathrin-coated vesicles, also contribute to the formation of the endosomal microdomains that facilitate cargo sorting. HRS contains a PSAP motif that binds to the ESCRT-I subunit TSG101, thereby recruiting the heterotetrameric ESCRT-I complex. ESCRT-I can recruit ESCRT-II, a heterotetramer consisting of two EAP20 subunits, one EAP30 subunit, and one EAP45 subunit. This recruitment likely occurs through an interaction between VPS28 and the GLUE domain of EAP45, which also serves as another PI3P- and ubiquitin-binding platform. The two EAP20 subunits of ESCRT-II directly interact with CHMP6 molecules (12), and ESCRT-I can also make direct contact with ESCRT-III through interactions between VPS28 and CHMP6 in their respective subcomplexes (13). This generates a nucleation complex that drives the polymerization of ESCRT-III filaments consisting mainly of CHMP4, along with CHMP2 and CHMP3. The ESCRT-III subunits interact with the endosomal membrane through clusters of basic residues in their core domain, myristoylation (in the case of CHMP6), or an N-terminal amphipathic helix (in the case of CHMP4) (14). Recent studies suggest that VPS4 also plays an active role in controlling neck constriction and vesicle scission (15).

### 2.2 Structures of VPS4

Vps4 enzymes play a crucial role in the ESCRT pathway by driving the exchange of subunits within ESCRT-III filaments and recycling them back into the cytoplasm using the energy of ATP hydrolysis (4). These enzymes are comprised of three distinct structural components (4): an N-terminal MIT domain, which binds the tails of ESCRT-III proteins; a central ATPase cassette includes large and small domains that facilitate tetramerization and ATP hydrolysis; and a β-domain insert located within the small ATPase domain, which binds an ATPase activator and ESCRT-III-binding protein-LIP5 (Vta1).

A study conducted in 2001 demonstrated that human cells express two non-allelic orthologs of the vacuolar protein sorting 4, namely hVPS4A and hVPS4B/SKD1, which share 80% identity and are involved in various intracellular protein trafficking processes (16). Both VPS4A and VPS4B utilize their microtubule interacting and transport (MIT) domains to bind to conserved sequence motifs located at the carboxy termini of the CHMP1-3 class of ESCRT-III proteins. The interaction between VPS4A/MIT-CHMP1A or VPS4B/MIT-CHMP2B complexes is reliant on this binding mechanism (17).

The active form of VPS4 is a hexamer complex that binds substrates in its central pore (18). By translocating ESCRT-III protein substrates through this pore, VPS4 unfolds them and drives membrane fission, ultimately leading to the recycling of ESCRT-III subunits (19). Any alterations to the structure or function of VPS4 protein could impact the membrane fission process mediated by ESCRT-III proteins and the subsequent recovery of ESCRT-III subunits.

### 2.3 Functions of VPS4

VPS4 ATPases play a crucial role in the ESCRT pathway by recognizing membrane-associated ESCRT-III assemblies and catalyzing their disassembly (20). During cytokinesis, the ESCRT pathway mediates the final membrane fission step of cytokinesis, which results in the permanent separation of newly formed daughter cells (21). The midbody adaptor protein CEP55 initiates cytokinesis by recruiting early-acting ESCRT factors ALIX and ESCRT-I (22). Subsequently, it promotes the recruitment and polymerization of critical ESCRT-II and ESCRT-III subunits, leading to the formation of filaments inside the midbody (23). These filaments, which are associated with the membrane, work in conjunction with the AAA ATPase VPS4 to constrict and cleave the midbody (24, 25).

ESCRTs bind to membranes, and influence their shaping, organization, properties and functions, either by binding to them directly or indirectly through other cytoskeleton elements (26). When binding with negatively charged membranes, the ESCRT-III components adopt an activated state that allows them to polymerize into filaments and spirals, and to interact with the AAA-ATPase Vps4 for polymer remodeling. Researchers Jukic et al. employed high-speed atomic force microscopy (HS-AFM) to study how the ESCRT-III proteins CHMP2A and CHMP3 facilitate membrane scission during cytokinesis. They suggested a model in which the CHMP2A-CHMP3 helices disassemble inside the cytokinetic necks, resulting in the constriction of the surrounding membrane tube and scission. In a study by Azad et.al, the process was observed in real-time using fluorescence microscopy and high-speed atomic force microscopy imaging. Their results confirmed the findings of Jukic et al. that CHMP2A-CHMP3 proteins play a crucial role in membrane tube constriction and scission during cytokinesis. CHMP2A-CHMP3-VPS4 is considered to be the minimum machinery necessary for membrane fission, which is of great importance in the formation of vesicles such as exosomes.

Moreover, VPS4 affects multiple cellular functions, including cell signaling, cell death, etc.

## 3 Possible mechanisms affecting carcinoma of VPS4

Recent research has highlighted the important role of VPS4 in cancer development and progression. Aberrant expression of VPS4 is associated with various types of cancer, including breast cancer, lung cancer, pancreatic cancer, etc, as **Supplementary Figures 1**, **2** show (27). Consequently, exploring the molecular mechanisms of VPS4-mediated cancer pathogenesis may pave the way for novel therapeutic approaches to cancer treatment.

### 3.1 VPS4 and cell division: cytokinetic abscission

The ultimate stage of cell division is cytokinetic membrane abscission (28), by which the cytoplasm of the parent cell is divided into two daughter cells. This process is controlled by a specialized organelle called the midbody (29), which forms at the site of cell division. Once the contractile ring has completed its constriction, the midbody (29) serves as a platform for the final abscission of the two daughter cells. This process is spatially and temporally regulated and requires the coordination of various proteins and pathways. The ESCRT pathway, especially the VPS4 protein, is essential for cytokinetic membrane abscission (30) and defects of VPS4 can lead to cytokinesis failure and the formation of multinucleated cells. In addition, abnormalities in VPS4 can lead to dysfunction of the checkpoint, resulting in erroneously replicated chromosomes still entering the daughter cells (31). Understanding the control of cytokinetic membrane abscission and the relevance of the VPS4 protein is critical for understanding basic cellular processes and developing innovative treatment techniques.

Cytokinetic abscission is influenced by checkpoints (32). ANCHR (Abscission/NoCut Checkpoint Regulator (33)) plays a critical role in regulating the abscission checkpoint, which serves to delay the abscission process in response to various mitotic issues, including incomplete nuclear pore reformation or chromatin bridges within the midbody. ANCHR achieves this regulation through its interactions with the most downstream component of the ESCRT machinery, namely the ATPase VPS4 (33). During cytokinesis, ANCHR and CHMP4C hold VPS4, which is capable of separating the two daughter cells, at the midbody ring until the abscission checkpoint signal is ended (34). When problems arise during mitosis, with the dephosphorylated of CHMP4C and assistance of other ESCRT-III-associated factors, the ANCHR-CHMP4C-VPS4 ternary complex is separated (4), and VPS4 is removed from the abscission sites. That results in the postponement of abscission (35).

Checkpoints function as DNA surveillance mechanisms that prevent the accumulation and propagation of genetic errors during cell division (36), whereas abnormalities or dysregulation of VPS4 implicate in the loss of the abscission checkpoint function, increasing the amount of mismatched DNA and allowing continuous cell division by compromising cells’ ability to exit the cell cycle. Cancer is associated with inadequate checkpoints, which allow substandard tumor cells to divide and grow (37).

### 3.2 VPS4 and cancer migration: through exosomes

Exosomes are small vesicles that contain a variety of bioactive substances, including DNA, RNA, and protein (38). Exosomes affect acceptor cells by interacting with extracellular receptors or being uptaken (39). Exosomes have been found to influence many biological processes through different molecular mechanisms, such as tumor immunity, tumor invasion, and metastasis (40). Vps4 collaborates with ESCRT-III to carry out specific membrane-remodeling actions that ultimately facilitate effective membrane scission during *in vivo* exosome biogenesis and recycle ESCRT-III subunits (11, 41). Jackson et al. reported the size and rate of formation of exosomes are regulated by Vps4 adenosine triphosphatase activity (42).

Exosomes have been identified as crucial mediators of intercellular communication in cancer, which ultimately leads to tumor progression. Furthermore, exosomes have also emerged as promising and progressing biomarkers for cancer (43). Cancer cells secrete extracellular vesicles that impact cancer progression by forming a tumor-promoting matrix and inducing fibroblast differentiation into cancer-associated fibroblasts (44). This differentiation depends on the triggering of alpha-smooth muscle actin expression and TGF-β signaling (45). Cancer-associated fibroblasts (CAFs) play a critical role in cancer invasiveness, and they also secrete extracellular vesicles that contribute to cancer cell invasiveness (46). As a result, extracellular vesicles released by cancer cells or CAFs have a direct influence on the matrix and other cells surrounding them, changing their functioning and driving cancer progression.

Recent studies have demonstrated that exosomes can impact metastasis by modulating the Epithelial-mesenchymal transition (EMT) and cancer stem cells (CSCs). Research by Lin H et all indicated (47), a decrease in miR-4454 can promote Vps4A and Rab27A expressions, which then induce exosome secretion and enhance the miR-4454 content in exosomes, thus accelerating the progression of liver carcinoma. EMT is considered a critical step in cancer cell metastasis (48). Han Q et al. discovered that Vps4A can mediate the PM localization and exosome release of β-catenin, consequently decreasing β-catenin signaling, and thereby inhibiting EMT and metastasis in HCC (49).

Exosomes secreted by cancer cells play an important part in the movement of cancer cells and the formation of premetastatic niches. These exosomes contain various molecules, including fibronectin, miRNA, proteases, and integrins, which can influence the extracellular matrix and facilitate cell migration and invasion. The fibronectin contained in these exosomes has been shown to be particularly important for cell migration (50). Tumor-derived exosomes can bind to individual components of the ECM, such as hyaluronic acid or laminin, and are rich in proteases that can degrade collagens, laminins, or fibronectin, leading to premetastatic niche preparation. These exosomes can also transfer metastatic capability between metastatic and nonmetastatic cancer cells (51). For example, extracellular vesicles containing miR-200, secreted by metastatic breast cancer cell lines, were shown to alter gene expressions and promote the mesenchymal-to-epithelial transition (MET) in nonmetastatic cells (52). Exosomes can also induce premetastatic niche formation in distant organs. Costa-Silva et al. found, in pancreatic ductal adenocarcinoma, exosomes were demonstrated to induce liver premetastatic niche formation in naïve mice. TGF-β secretion and fibronectin upregulation in recipient hepatic cells create a fibrotic microenvironment, while the macrophage migration inhibitory factor (MIF) contained within exosomes counteracts bone-marrow-derived macrophages leading to metastasis (53). Exosomal integrins also play a crucial role in determining organ-specific metastasis. Integrins are cell surface receptors that mediate cell adhesion and signaling, integrins α6β4 and α6β1 were associated with lung metastasis, and integrin αVβ4 was linked with liver metastasis (54).

Sylvain Loric et al. proposed that exosomes play an important role in the formation of mammary stem cells (MaSCs), which are probable candidates for breast cancer initiation (55, 56). In addition, Exosomes mediate epithelial-mesenchymal transition and the formation of cancer stem cells, playing a crucial role in tumor metastasis. Furthermore, exosomes can influence the extracellular matrix and facilitate cell migration and invasion. Stefańska et al. indicated (46) in their review that Exosomes can also induce immune suppression or promote tumor progression by affecting immune regulation and extracellular angiogenesis. VPS4 induces exosome formation and is vital in CSCs and cancer cell migration. Overall, the study of exosomes and their roles in cancer metastasis is an active area of research and has the potential to provide new insights into cancer biology and therapy.

### 3.3 Synthetic lethality between VPS4A gene and VPS4B gene

Synthetic lethality was first reported in 1968, which refers to the phenomenon that the simultaneous inactivation of two non-lethal genes will lead to cell death. At present, poly (ADP-ribose) polymerase inhibitors (PARPi) operate through a “synthetic lethality” mechanism with mutant DNA repair pathways genes in cancer cells, and PARPis are widely used in cancer such as ovarian cancer (57). There are two forms of VPS4 in the human body and recently it is confirmed that VPS4A and VPS4B are essential enzymes for the ESCRT pathway and have no substitution (58), loss of both is fatal (59).

VPS4B expression was significantly downregulated in colorectal cancer (CRCs) (60). Sheffer et. al (61) found that in immunocompromised NU/J mice, injection of HCT116 cells with the knockout of VPS4B and doxycycline (Dox)‐inducible VPS4A‐targeting shRNA expression (HCT116 VPS4B −/− shVPS4A) inhibits tumor growth in mouse. Neggers et al. also reported (62) that induction of VPS4A suppression in human VPS4Bloss SMSCTR (rhabdomyosarcoma) and SNU213 (pancreatic ductal adenocarcinoma) cancer cells result in near-complete tumor regression (SMSCTR) or potent tumor growth inhibition (SNU213) and improved survival in both models. They confirmed that suppression of VPS4A in VPS4B-deficient cells leads to selective accumulation of ESCRT-III filaments, resulting in cytokinesis defects, nuclear deformation, G2/M arrest, apoptosis, and significant tumor regression. In summary, there exists “synthetic lethality” of the VPS4A gene and VPS4B gene in cancer, especially in CRCs.

It is found that the ESCRT ATPases VPS4A and VPS4B score as strong synthetic lethal dependencies. VPS4A is essential in cancers harboring loss of VPS4B adjacent to SMAD4 on chromosome 18q and VPS4B is required in tumors with co-deletion of VPS4A and CDH1 (E-cadherin) on chromosome 16q (62). As a result, VPS4A and VPS4B may become high-priority therapeutic targets for malignancies characterized by 18q or 16q deletion. However, there is no experimental evidence that VPS4B depletion causes cell death in tumors with low or absent VPS4A levels.

### 3.4 VPS4 and signaling pathways

The role of Vps4 has been studied in various cellular signaling pathways, including the Wnt pathway. Rodahl et.al (63) reported that double deficiency in dVps4 and JNK signaling leads to the formation of neoplastic tumors in drosophila. Wnt signaling is one of the key cascades regulating development and stemness and is proven to be tightly associated with cancer (64). Typical Wnt signaling requires inhibition of Glycogen Synthase Kinase 3 (GSK3) activity (3). Taelman et al. reported that Wnt signaling triggers the sequestration of GSK3 from the cytosol into multivesicular bodies (MVBs), so that this enzyme becomes separated from its many cytosolic substrates (65). Furthermore, they investigated the role of Vps4 in the Wnt signaling pathway. A point mutation in the ATPase site of VPS4 (Vps4-EQ), cause a potent dominant-negative form that inhibited the formation of intraluminal vesicles and blocked Wnt3a signaling. They also tested the requirement of the ESCRT machinery for axis induction by Siamois, a homeobox gene activated by Wnt signaling, and found that Vps4-EQ mRNA was unable to inhibit Siamois secondary axes. This suggests that Vps4 is important not only for GSK3 sequestration but also for other downstream events in the Wnt signaling pathway. Overall, Vps4 plays a crucial role in the Wnt signaling pathway and could have implications for understanding diseases associated with disrupted Wnt signaling.

### 3.5 VPS4 and cell death: pyroptosis and ferroptosis

Cancer cells often have defects in cell death executioner mechanisms, which is one of the main reasons for therapy resistance. How to effectively induce cancer cell death, including cancer cell pyroptosis (41), ferroptosis, etc., has become a focus in the development of anticancer drugs. Many cells need to complete a set of effector programs before they die, which dependents on the ESCRT-drive membrane repair to allow cells to complete the programs before they die (66). ESCRT-III components primarily play a role in repairing damage to the plasma membrane and maintaining cell survival before cell lysis (67). VPS4, as the last step of the ESCRT pathway, plays important roles in various cellular death processes, and dysregulation or deficiency of VPS4 can affect cell death to varying degrees.

Pyroptosis is a form of regulated necrosis induced by the pore-forming protein gasdermin D (GSDMD) that damages the plasma membrane. Nara et al. proposed that during pyroptosis, after cytosolic caspases cleave GSDMD to form nanoscale membrane pores, CHMP4B is recruited to the plasma membrane and clusters around the neck to remove the GSDMD pores and preserving plasma membrane integrity, thus limiting proinflammatory cytokine interleukin-1β (IL-1β) and IL-18 release through GSDMD pores to inhibit pyroptosis (67). VPS4B ATPase is activated to dismantle the ESCRT-III complex after membrane scission (68).

Cancer cells exhibit an increased iron demand to enable growth compared with normal cells. This iron dependency can make cancer cells more vulnerable to iron-catalyzed necrosis, referred to as ferroptosis (69). As reported, the membrane damage caused by ferroptosis stimulus can be repaired by ESCRT-III-dependent membrane scission machinery (7, 70), Dai et al. also suggested that ESCRT-III confers resistance to ferroptosis cell death, allowing cell survival under stress conditions (71). If VPS4 is abnormal or dysfunctional, it could potentially disrupt the ESCRT pathway, leading to a reduced ability to repair the membrane damage and an increased likelihood of ferroptosis in cancer cells.

Dysregulation or deficiency of VPS4 can affect various cellular death processes, including pyroptosis, and ferroptosis. Additionally, dysfunctional VPS4 can reduce the ability to repair membrane damage, increasing the likelihood of ferroptosis in cancer cells. Defects in Vps4 can also affect pyroptosis, suggesting that targeting VPS4 could be a potential strategy for inducing cancer cell death.

### 3.6 VPS4 and Autophagy

Autophagy is a cellular mechanism in which the cell “self-eats” misfolded proteins and dysfunctional organelles to autophagosomes (APs) and subsequently deliver them to lysosomes for degradation (72). The study found that ESCRTs repair small lysosomal membrane pores by direct membrane sealing (73). Autophagosome biogenesis has a close relationship with ESCRTs (74), including VPS4. Defects in the fusion of APs and lysosomes are associated with Vps4 mutants in human cells (75). SKD1 is a member of the family of ATPases associated with cellular activities. Fujita et al. described that when a mutant of SKD1 that lacks ATPase activity [SKD1(E235Q)] was overexpressed in mammalian cells will cause an accumulation of basolateral recycling receptors, SKD1 regulates multiple steps of membrane transport out of early endosomes and the reformation of lysosomes from a hybrid organelle (76). It has been proven that the function of Vps4 in maintaining axonal autophagy is conserved in mammals, and Vps4 is essential and sufficient to promote autophagic flux (77).

VPS4 plays a crucial role in various cellular processes such as endosomal sorting, membrane trafficking, cytokinesis, and cell signaling transduction. Recent research has highlighted the significance of VPS4 dysregulation in cancer development and progression. Targeting the synthetic lethality of VPS4A and B genes may provide a novel therapeutic strategy for cancer treatment. *In vitro* and *in vivo* studies have shown that inhibiting VPS4 can reduce cancer cell migration and invasion. In conclusion, VPS4 plays a crucial role in various cellular processes, and its dysregulation has been linked to cancer development and progression.

## 4 Application of VPS4 in carcinoma

Research has shown that VPS4 expression levels are often altered in various types of cancer, and this dysregulation can have significant effects on tumor development and progression. In addition, VPS4 has been proven to affect a range of cellular processes, which are important for tumor cell survival and growth. Current research shows that VPS4 is related to tumor staging, prognosis, and treatment, and further relationships need to be explored.

### 4.1 VPS4B and Staging

The expression levels of VPS4B in different types of cancer have been shown to be associated with varying clinical and pathological factors, as well as with patient survival outcomes. Lin et al. conducted that there is a negative correlation between VPS4B expression and EGFR abundance in breast tumors, and high-grade or recurrent breast carcinomas have decreased levels of VPS4B expression, which indicates that VPS4B may have a tumor-suppressive role in breast cancer (78). Lin et al. conducted that in 2D and 3D culture systems of EGFR/HER2-expressing SKBR3 breast cancer cells whose VPS4B is selectively downregulated under hypoxic conditions, EGF-induced EGFR degradation is attenuated. EGFR signaling was responsible for cell growth, invasion, and metastasis in breast cancer (79). There is a negative correlation between VPS4B expression and EGFR stability in breast tumors (78). VPS4B is also positively associated with pancreatic cancer development. Transplantation of VPS4B-deficient pancreatic tumors into immune competent mice impairs autophagy and resulting in increased accumulation of CD8 T cell-derived granzyme B and tumor cell lysis (80).

In NSCLC, VPS4B showed high expression and a significant correlation with tumor size, histological differentiation, clinical stage, and Ki-67. Experimenters found, knocking down the expression of VPS4B (81) and analyzing the proliferation of A549 NSCLC cells *via* Western blot, CCK8, and flow cytometry assays indicate that loss of VPS4B could inhibit cell cycle progress and abolish the proliferation of A549 cells (82).Correspondingly, Y. Liu et al. also confirmed that knocking down VPS4B led to cell cycle arrest and reduced cell proliferation of HCC cells (82).

In summary, high expression of VPS4B is associated with tumor proliferation and poor prognosis, suggesting that VPS4B may become an important assessment factor in tumor staging.

### 4.2 Prognosis

As mentioned in **3.1**, VPS4B is associated with the prognosis of various tumors. In order to have a further understanding of the relationship between the expression of the VPS4 gene and tumor, we downloaded the unified and standardized pan-cancer data set from the UCSC (83) database: TCGA TARGET GTEx (PANCAN, N=19131, G=60499), and further we extracted ENSG00000132612 (VPS4A) and ENSG00000119541(VPS4B) gene expression data in each sample. In addition, we also obtained from the TCGA prognosis study (84) previously published on Cell obtained a high-quality TCGA prognosis data set, obtained TARGET follow-up data from UCSC’s cancer browser (83) as a supplement, and excluded samples whose follow-up time was less than 30 days. Expression data and disease-specific survival data of corresponding samples are as follows (27) (**Supplementary Figure 1**, **2**).

There is evidence to suggest that high expression of VPS4 may be associated with poor prognosis in certain diseases. It is important to note, however, that the relationship between VPS4 expression and prognosis is likely to be complex and may vary depending on the specific disease and context. Further research is needed to fully understand the role of VPS4 in disease progression and its potential as a therapeutic target.

### 4.3 Feasibility of application in therapy

So far, exosomes are emerging as promising new carriers for drugs and biotherapeutics in glioblastoma (85, 86). By combining exosome research with nanotechnology, exosome-like systems can be developed as a competitive approach for innovative targeted anti-cancer therapies (87). Moreover, exosomes contain microRNAs, proteins, and other biomolecules which reflect the physiological state and pathological characteristics of secretory cells. The use of exosomes as biomarkers is a promising approach in the era of liquid biopsy, especially in NSCLC (88) and glioma (89).

VPS4A and VPS4B are vesicle-fusing ATPases, which belong to the AAA-type (ATPase associated with a variety of cell activities) ATPase superfamily and promote the reaction of hydrolysis of ATP in the positive progress. The majority of AAA ATPases are singular (type I) or dual-looped (type II) homo-hexamers as their operative entities. The VPS4A/B end lysosomal sorting ATPase is typical type I (90). Specific AAA ATPase inhibitors are necessary in several well-studied forms of AAA ATPases due to structural differences (91). Drugs that operate directly on VPS4 are still under investigation. To learn more about the druggability of VPS4 proteins, we searched a public cancer comprehensive knowledge base canSAR(https://cansar.ai/) and found that VPS4A and VPS4B have druggable structures or enzymes, which are listed in **Table 1** (92).

**Table 1:** The druggable structures of VPS4A and VPS4B screened by the can SAR database.

	Compound	Name	Ligand efficiency	Bioactivity Type
VPS4A	3450612	(S)-2-amino-N-(5-(6-chloro-5-(phenylsulfonamido)pyr idin-3-yl)-4-methylthiazol-2-yl)-3-methylbutanamide;	0.12	IC50 280 nM
VPS4B	3446053	NMS694; canSAR3446053	0.14	IC50 260 nM
	3446029	NMS-485A; canSAR3446029	0.15	IC50 500nM
	3231320	canSAR3231320	0.1	IC50 710nM

VPS4A has a predicted structure in the alpha-fold database, which means 3d-based ligandability has been assessed and available. There are 3 chains 3D Structure of VPS4A, and experimental structural coverages 77 positions of 3 chains (93). Ligandable cavities of VPS4B are primarily in the ATPase family associated with various cellular activities (AAA) domains.

In general, VPS4A and VPS4B druggability is an attractive field of research, and it will be interesting to see how these proteins can be targeted for drug development.

## 5 Conclusion

VPS4 is critical for tumor biology through its roles in cell division, cell metastasis, cell death, signaling induction, etc. These functions of VPS4 make it a potential target for cancer diagnosis and treatment. As an important link in the formation of MVB, VPS4 may have a close relationship with the tumor microenvironment and immunomodulation, which is not confirmed yet. Moreover, VPS4B regulates apoptosis of chondrocytes *via* p38 Mitogen-Activated Protein Kinases (MAPK) in osteoarthritis (94) and Crohn’s disease (95), providing a possible pathway by which VPS4 series proteins affect tumors. Therefore, continued in-depth investigation is required. In conclusion, a thorough understanding of VPS4 will improve cancer clinical translational potential.

## Author contributions

LH and YY analyzed and interpreted the data. SZ collected information. LH, YY and SZ worked equally as major contributors in writing the manuscript. All authors contributed to the article and approved the submitted version.

## Supplementary material

Supplementary Figure 1 VPS4A expression in different cancers

Supplementary Figure 2 VPS4B expression in different cancers

Supplementary Figure 3 VPS4A expression and disease-specific survival in cancer

Supplementary Figure 4 VPS4B expression and disease-specific survival in cancer
