# Supplementary figures and images for: The role of Vps4 in cancer development
Source: Front Oncol. 2023 Jun 19;13:1203359. doi: 10.3389/fonc.2023.1203359 (PMC10315677; doi:10.3389/fonc.2023.1203359)

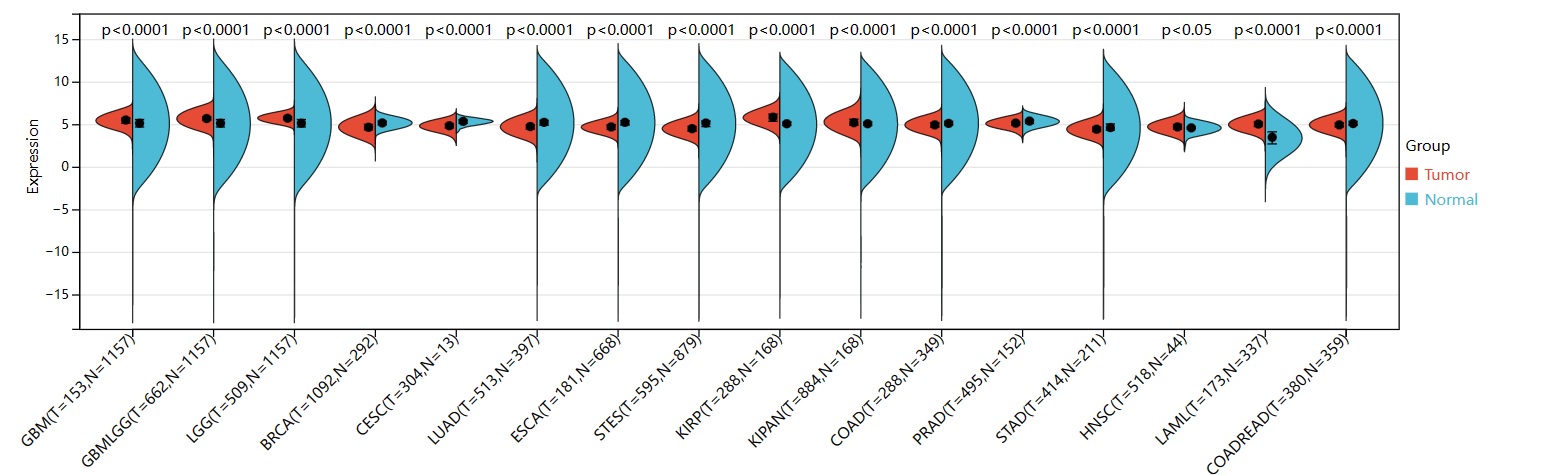

Supplement: Supplementary Figure 1 — VPS4A expression in different cancers [file Image_1.jpeg]

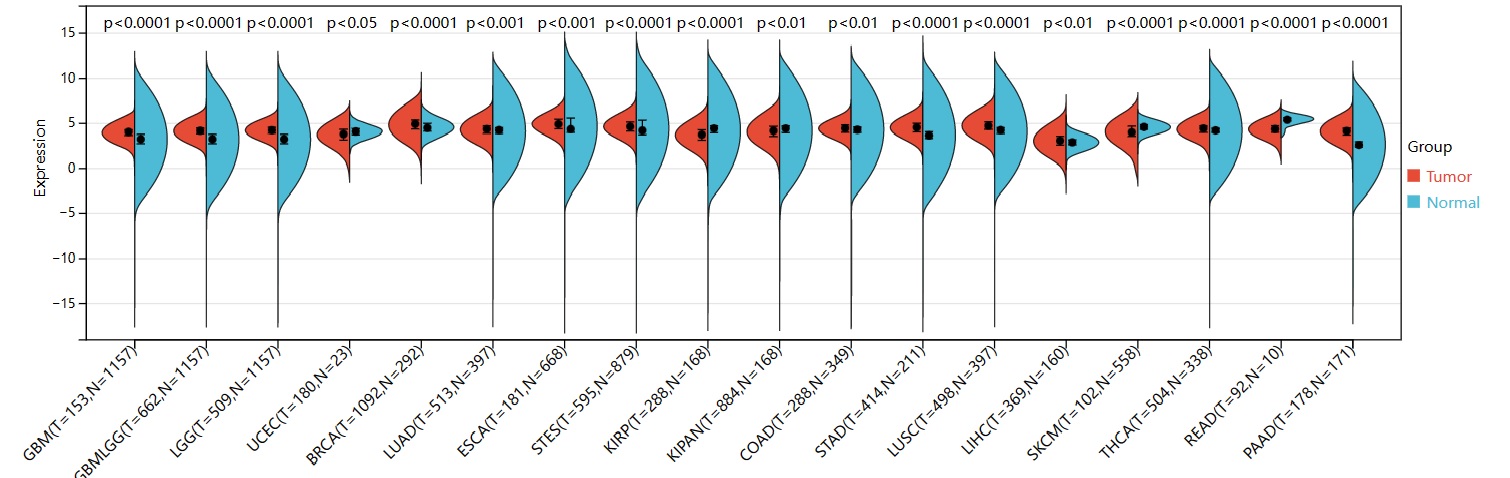

Supplement: Supplementary Figure 2 — VPS4B expression in different cancers [file Image_2.jpeg]

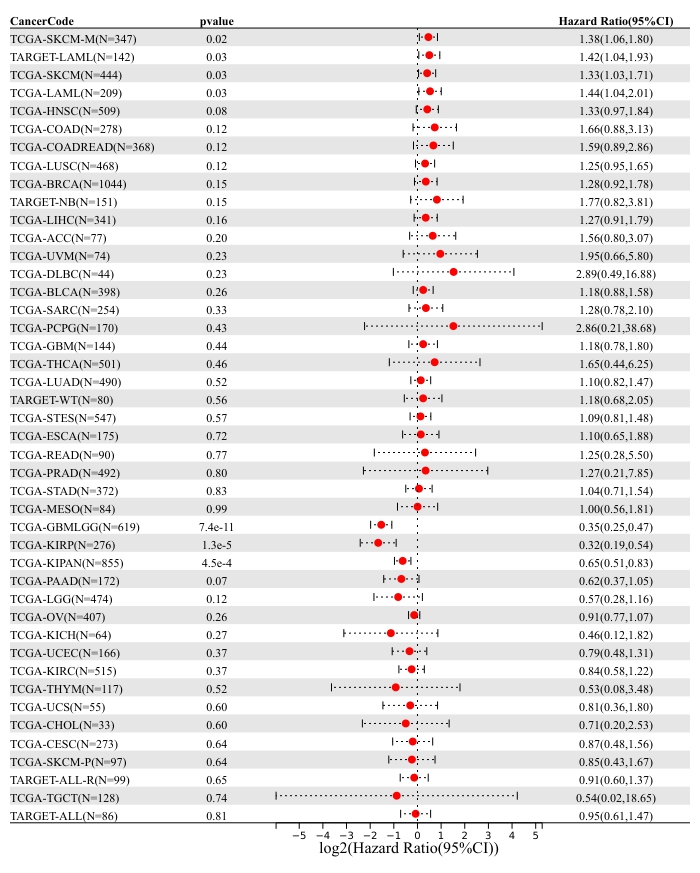

Supplement: Supplementary Figure 3 — VPS4A expression and disease-specific survival in cancer [file Image_3.jpeg]

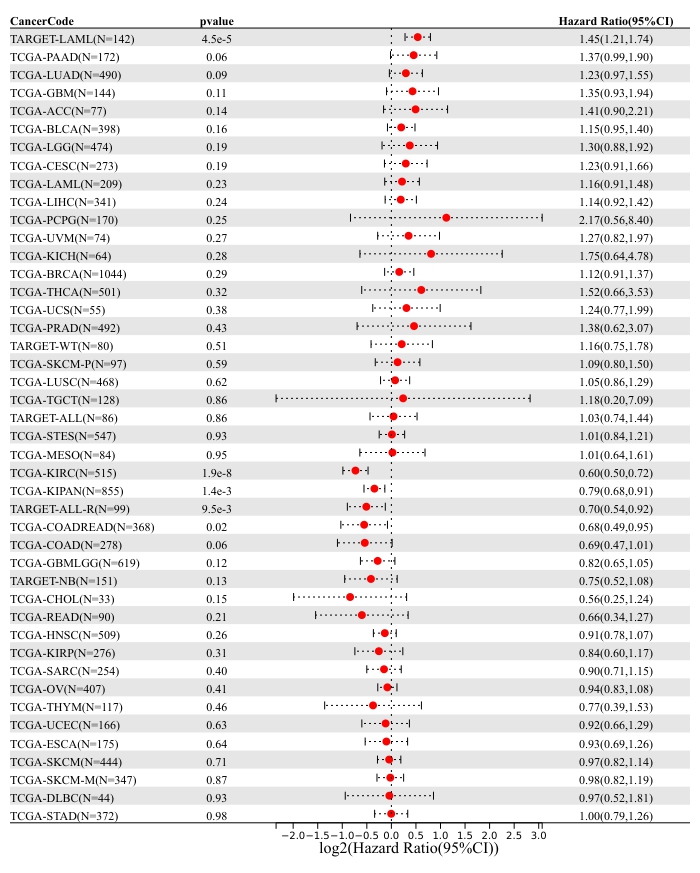

Supplement: Supplementary Figure 4 — VPS4B expression and disease-specific survival in cancer [file Image_4.jpeg]
